# Functional Properties of Gelatin–Alginate Hydrogels for Use in Chronic Wound Healing Applications

**DOI:** 10.3390/gels11030174

**Published:** 2025-02-27

**Authors:** Olha Maikovych, Pamela Pasetto, Nataliia Nosova, Olena Kudina, Dmytro Ostapiv, Volodymyr Samaryk, Serhii Varvarenko

**Affiliations:** 1 Department of Organic Chemistry, Lviv Polytechnic National University, 12 Bandera Str., 79013 Lviv, Ukraine; nataliia.h.nosova@lpnu.ua (N.N.); volodymyr.y.samaryk@lpnu.ua (V.S.); serhii.m.varvarenko@lpnu.ua (S.V.); 2 Institut des Molécules et Matériaux du Mans, Faculté des Sciences & Techniques, Le Mans Université, Avnue Olivier Messiaen, 72085 Le Mans, Cedex 09, France; pamela.pasetto@univ-lemans.fr; 3 CÚRAM Research Ireland Centre for Medical Devices, University of Galway, H92 W2TY Galway, Ireland; olena.kudina@universityofgalway.ie; 4 Institute of Animal Biology NAAS, 38 V. Stusa Str., 79034 Lviv, Ukraine; oddost@ukr.net

**Keywords:** hydrogel, gelatin, sodium alginate, crosslinking, wound dressing, drug delivery

## Abstract

In this study, a hydrogel material based on porcine gelatin and sodium alginate was synthesized for use as a dressing for chronic wound treatment. The hydrogels were covalently cross-linked using polyethylene glycol diglycidyl ether (PEGDE 500), and the interaction between the components was confirmed via FTIR. The properties of the resulting hydrogels were examined, including gel-fraction volume, swelling degree in different media, mechanical properties, pore size, cytotoxicity, and the ability to absorb and release analgesics (lidocaine, novocaine, sodium diclofenac). The hydrogel’s resistance to enzymatic action by protease was enhanced both through chemical cross-linking and physical interactions between gelatin and alginate. The absorption capacity of the hydrogels, reaching 90 g per dm^2^ of the hydrogel dressing, indicates their potential for absorbing wound exudates. It was demonstrated that the antiseptic (chlorhexidine) contained in the structured gelatin–alginate hydrogels can be released into an infected substrate, resulting in a significant inhibition of pathogenic microorganisms (*Escherichia coli*, *Staphylococcus aureus*, *Pseudomonas aeruginosa*, and *Aspergillus niger*). These results clearly demonstrate that the obtained hydrogel materials can serve as non-traumatic dressings for the treatment of chronic and/or infected wounds.

## 1. Introduction

Chronic wounds, including diabetic ulcers, pressure ulcers, and burns, pose a serious health problem worldwide [1,2]. Effective wound care requires modern materials that can create a favorable environment for wound healing while protecting the wound from external irritants and contaminants.

All types of wounds (acute and chronic) require quality clinical care to prevent delayed healing, which can be caused by microbial infections and other adverse factors. Over 300 types of wound dressings are available on the market; however, the same dressing cannot be used for treating all wound types [3]. The global market for chronic wound treatment was valued at USD 11.61 billion in 2021. It is expected to grow from USD 12.36 billion in 2022 to USD 19.52 billion by 2029, demonstrating a CAGR of 6.7% during the forecast period [4].

The development of new drug forms with an enhanced bioavailability and prolonged, controlled drug release is highly relevant, especially when these forms are convenient to synthesize and use. The development of new drug forms with immobilized drugs is expanding. Hydrogels have become promising candidates for wound dressings due to their ability to maintain a moist environment, facilitate gas exchange, and support tissue regeneration [5,6,7]. They are used to make contact lenses, membranes, biosensors, materials for artificial tissues (such as skin and cartilage), and drug delivery systems [8,9]. Polymers are typically used as carriers, and biopolymers are the most desirable in this regard [10]. For effective wound treatment, biopolymer-based hydrogels are considered modern materials that can create a favorable healing environment while simultaneously protecting the wound from external irritants and contaminants.

Since the structure of hydrogels is similar to the extracellular matrix of many tissues, they can fill wounds, absorb exudates, and deliver drugs without causing additional damage, thus promoting an accelerated tissue regeneration [10,11].

Another advantage is the possibility of designing hydrogels with a high bioavailability and minimal immune response. The biomimetic nature of hydrogels, and their ability to maintain a moist environment for wound healing and skin regeneration are highly beneficial for achieving positive treatment outcomes, especially in cases of burn wounds [12,13]. The incorporation of antimicrobial agents and various biological molecules into hydrogels enables their use as dressings for the effective treatment of chronic/infected wounds [14,15]. The development of ideal wound dressing materials with desirable antibacterial and good wound healing properties remains a major challenge affecting the regeneration of bacterially infected wound tissues [16].

Hydrogel materials often exhibit insufficient strength and a limited release of hydrophobic molecules. Their ability to be modified can expand the potential applications of such materials. As noted in [17], incorporating a hydrophobic component imparts unique amphiphilic properties to hydrogels. The design of an amphiphilic inter-penetrating network can be extended to other polymeric hydrogel systems, opening new opportunities for drug delivery and the development of smart materials.

In [18], the possibility of modifying cyclodextrin-based hydrogels for the hydrophobic delivery of baicalein was demonstrated, showing potential for biomedical applications, particularly as a controlled delivery system for hydrophobic drugs.

Previously, in [19], the development of resorbable and biodegradable enzyme-crosslinked gelatin–alginate semi-IPN hydrogel dressings with curcumin was proposed. Moreover, in [20], a method for creating gelatin–alginate hydrogel with adipose-derived stem cells for skin regeneration was described, utilizing crosslinking in a 5% CaCl_2_ solution. However, these methods have certain limitations and are rather time-consuming due to the multistep process required for obtaining hydrogel materials.

In recent years, hydrogels based on natural polymers such as gelatin, pectin, chitosan, and sodium alginate have attracted considerable attention in wound care. Gelatin, derived from collagen, has excellent biocompatibility and biodegradability, making it suitable for medical use [21,22]. Sodium alginate, derived from seaweed, has unique gel-forming properties and has been widely studied for its wound-healing potential [23,24].

The purpose of this article is to describe the synthesis and characterization of gelatin and sodium alginate hydrogels crosslinked with polyethylene glycol diepoxide as a basis for wound dressings. We will discuss the physicochemical properties of these hydrogels, including their swelling behavior, mechanical strength, and degradation kinetics, which are crucial factors affecting their effectiveness as wound dressings.

In addition, we will study the conditions of the saturation and release from gelatin–alginate hydrogels of a number of drugs, such as novocaine, lidocaine, diclofenac sodium, and chlorhexidine, which are used to increase the therapeutic effectiveness of hydrogels, due to their anesthetic effect and antiseptic properties [25,26].

To summarize, hydrogels based on gelatin and sodium alginate hold great promise as versatile wound coverage platforms [27,28,29]. With a full understanding of their properties and efficacy in wound healing applications, we can pave the way for the development of advanced biomaterials that meet the needs of treating hard-to-heal and chronic wounds and improve patient care [30,31,32,33].

## 2. Results and Discussion

### 2.1. The Synthesis Characteristics of Gelatin–Alginate Hydrogels

A recurring challenge for hydrogels is their low mechanical strength, which has significantly limited their applications, especially in the medical field. Researchers have been working to improve this since the development of hydrogels [34,35,36]. There are two main strategies for improving the mechanical properties of hydrogel materials. The first approach involves producing multimodified materials, such as double networks, double-crosslinked networks, and interpenetrating networks. The second strategy is to incorporate reinforcing elements, including fibers, meshes, and nonwoven polypropylene materials [37,38,39].

**Figure 1:**
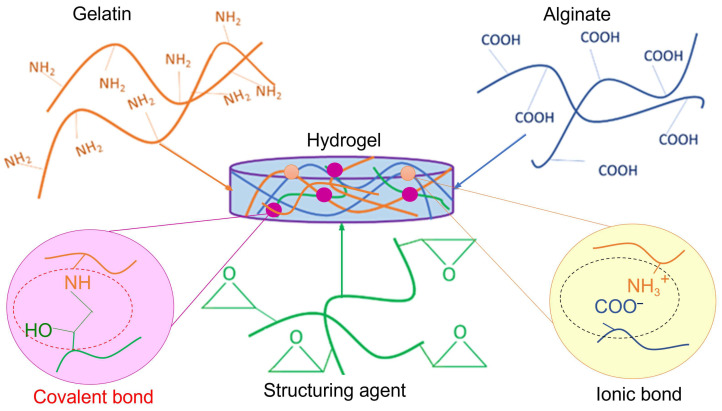
A scheme of the combined gelatin–alginate hydrogel synthesis.

This paper focuses on the preparation of hydrogels based on gelatin and sodium alginate using a diepoxy crosslinker derived from polyethylene glycol 400 as a structuring agent. Mechanical properties were enhanced by maximizing the number of crosslinking points within the hydrogel structure. The synthesis scheme is shown in Figure 1. Gelatin–alginate hydrogel samples were prepared at varying crosslinker-to-(gelatin–alginate) ratios, ranging from 1:1 to 1:50, as described in the experimental section, and their properties are summarized in Table 1.

The structure of hydrogels is formed due to several factors of its spatial network formation [40,41]. In particular, gelatin complexes with ionic polysaccharides are primarily formed by intermolecular electrostatic interactions and hydrogen bonds [42,43]. Ionic interactions of the complementary functional groups allow fragments of gelatin macromolecules to bind to sodium alginate macromolecules. The formation and stability of such polyelectrolyte complexes depend on various factors, including the ionization degree of polyelectrolytes, the charge distribution along the polymer chains, the nature and position of ionic groups, the flexibility of the polymer chain, and the molecular weight and ratio of polyelectrolytes, as well as the temperature, ionic strength, and pH of the reaction medium [44]. Under specific conditions—such as certain ratios of biopolymers, their concentration, pH, and ionic strength of the medium, these complexes can form hydrogels independently [45,46,47].

**Figure 2:**
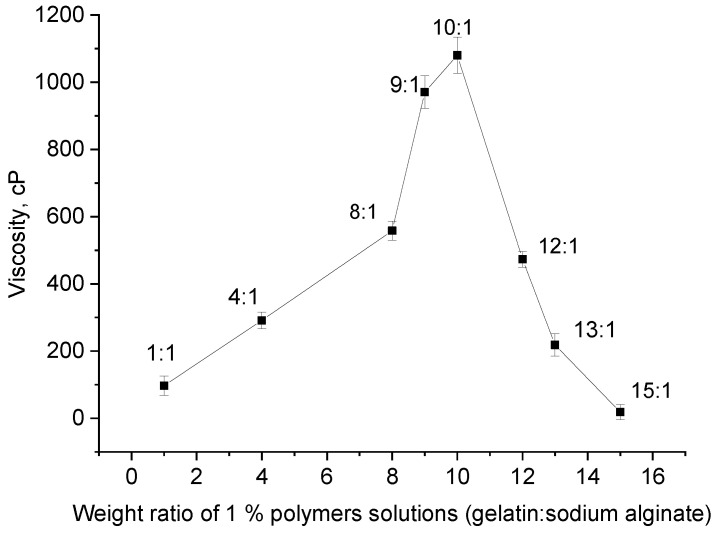
The viscosity of a mixture of 1% aqueous polymer solutions vs. their ratio.

We propose to increase the number of crosslinks by structuring a mixture of biopolymers that have already formed polyelectrolyte complexes (PECs) using a diepoxy crosslinker based on polyethylene glycol 400 (PEGDE 500). Previous studies have demonstrated PEGDE 500’s ability to crosslink gelatin macromolecules to form hydrogels [25,26]. To implement this approach of creating intermolecular bonds in the mixture, it was crucial to identify the optimal range of biopolymer ratios at which a water-soluble PEC is formed. Such a PEC can be considered as a high molecular weight polymer, of whose further structuring can lead to hydrogels with improved properties. In this case, the change in the viscosity of the biopolymer solution serve as a qualitative and quantitative indicator of the adduct formation. The formation of the optimal number of intermolecular bonds, at which the PEC macromolecules remain in the most possible unfolded state in the solution, depends specifically on the ratio of the two polymers. Data shown in Figure 2, which illustrate the results of the rheological studies of 1% aqueous solutions of the polymer mixture at different ratios, indicate a significant increase in viscosity within the gelatin–alginate ratio range of 8:1 to 12:1. The analysis of this section reveals that the maximum viscosity, nearly 10–12 times higher, occurs at a gelatin–alginate ratio of 10:1. The established ratio was used in further hydrogel syntheses to investigate the effect of the covalent structuring of this polymer complex using the diepoxy crosslinker PEGDE 500.

An essential factor in hydrogel synthesis is the total polymer content, as it directly affects their properties. In a previous study of gelatin hydrogels obtained using PEGDE 500, the optimal total polymer concentration of 18% was found [25]. At lower concentrations, the spatial structure of the hydrogel was not formed, and samples of an unsatisfactory quality were obtained. At higher concentrations, no significant advantages were achieved. For gelatin–alginate hydrogels, a polymer concentration above 18% led to a rapid increase in solution viscosity and gelation occurs, hindering the removal of air bubbles and mixing homogeneity. The studies showed that the lowest limit of polymer concentration, at which gelation occurred, is 12%, and this concentration was selected for comparison in further studies. This fact alone favorably distinguishes the new hydrogels from the previously studied gelatin hydrogels, where gelation occurred at a total content of at least 18% of the polymer.

Hydrogel samples were synthesized at the reaction mixture pH of 5.5–6.0, which was achieved spontaneously without additional pH regulators. Under these conditions, hydroxyl and carboxyl groups from both gelatin and sodium alginate most likely do not react with epoxy groups. Structuring occurs mainly through the interaction of the structuring agent with the lysine amino group in the gelatin macromolecules [25].

**Figure 3:**
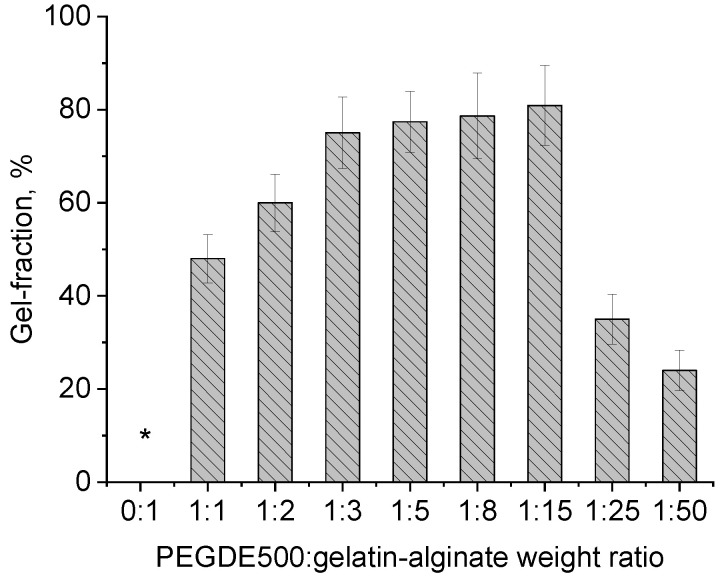
Gel-fraction values of the hydrogel obtained at different PEGDE 500/gelatin–alginate weight ratios. (*—unstructured gelatin-sodium alginate mixture).

The gel-fraction value for hydrogels obtained in different contents of the structuring agent indicates that under certain synthesis conditions and ratios in the range of 1:2–1:15, the maximum number of crosslinks is achieved resulting in a hydrogel that maintains stable forms when heated to 50 °C. Unlike the sample of the unstructured gelatin–sodium alginate mixture, the obtained hydrogel swells in water but does not dissolve (Figure 3).

Based on the above-described data, synthesis conditions were selected to maximize the involvement of reagents in the creation of a three-dimensional polymeric hydrogel network.

### 2.2. The Chemical Characterization of Gelatin–Alginate Hydrogel

**Figure 4:**
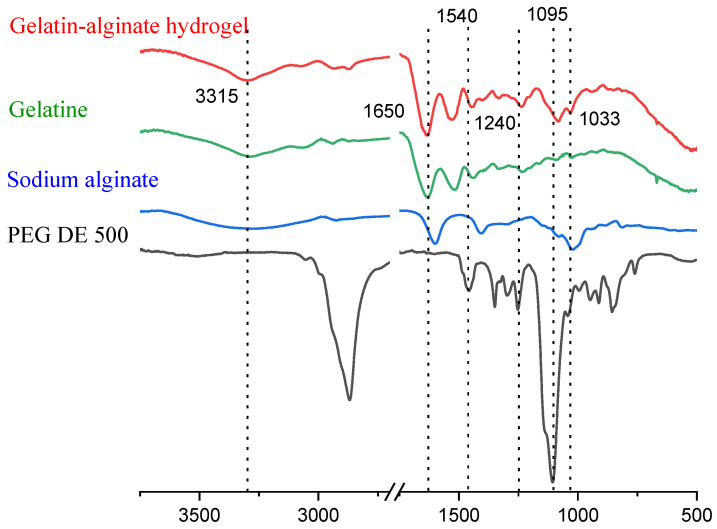
The FTIR spectra of the PEGDE 500, alginate, gelatin, and gel fraction of the reaction product isolated after structuring gelatin–alginate.

Figure 4 shows the infrared spectra of the dried samples of gelatin, sodium alginate, PEGDE 500 structuring agent, and the polymer of the hydrogel gel fraction. The absorption peaks at 1650 cm^−1^ and 1540 cm^−1^ in the infrared spectrum of pure gelatin corresponding to the stretching vibrations of C-O and C-N (amide I band) and the bending vibrations of the -NH group (amide II band), respectively. For sodium alginate, the characteristic absorption bands at 1616 and 1419 cm^−1^ correspond to the peaks of the asymmetric and symmetric stretching of -COO-, respectively. In addition, the bands at 1300 cm^−1^ (C-O stretching), 1086 cm^−1^ (mannuronic units), 1033 cm^−1^ (guluronic units), and 817 cm^−1^ (α-configuration of the guluronic units) also relate to the structure of the saccharide. In the infrared spectrum of the structured polymer network (a washed hydrogel, devoid of ungrafted fragments), the absorption peaks at 1616 cm^−1^ and 1419 cm^−1^ of the sodium alginate became less prominent and overlapped with the peaks of pure gelatin. Since the formation of strong hydrogen bonds leads to a shift in the peaks in the IR spectra due to a change in the electron density at the hydrogen bond site, we believed that hydrogen bonds were formed between the chains of gelatin and sodium alginate.

The absorption band at 1095 cm^−1^ corresponds to the vibrations of the C-O-C groups in PEGDE 500, which is absent in the spectrum of both gelatin and sodium alginate. The absorption band of guluronic residues at 1033 cm^−1^ in the spectrum of sodium alginate (not present in gelatin and the structuring agent) also appears. A peak at 3315 cm^−1^ assigned to -N-H stretching and hydrogen bonding (amide A), stretching vibrations of the gelatin amide group carbonyl (C=O) at 1650 cm^−1^ (amide I), and deformational vibrations of the -N-H group at 1540 cm^−1^ (amide II) remained present in the spectrum, with only a slight change in the ratio of functional groups compared to the intact gelatin involved in the reaction. The spectrum of the obtained hydrogel shows no asymmetric vibrations of the C-O bond stretching in the oxirane ring at 1240 cm^−1^, associated with its opening. This indicates a chemical modification of the oxirane groups due to their reaction with gelatin amino groups and a change in their chemical environment in the structured hydrogel matrix. This serves as evidence of the effectiveness of covalent crosslinking between the components. This suggests that the three-dimensional structure of the polymer consists of gelatin macromolecules crosslinked by a diepoxide crosslinker and is connected with alginate macromolecules through hydrogen and ionic interactions.

### 2.3. The Mechanical Properties of Gelatin–Alginate Hydrogels

The study on mechanical properties was conducted by measuring the force in cylindrical samples of hydrogels with a diameter of 10 mm under uniaxial compression. Since the lowest values were obtained for the gelatin hydrogel, which was destructed at a 4.5 mm strain, the values for other hydrogel compositions are given at 4.5 mm of strain.

**Figure 5:**
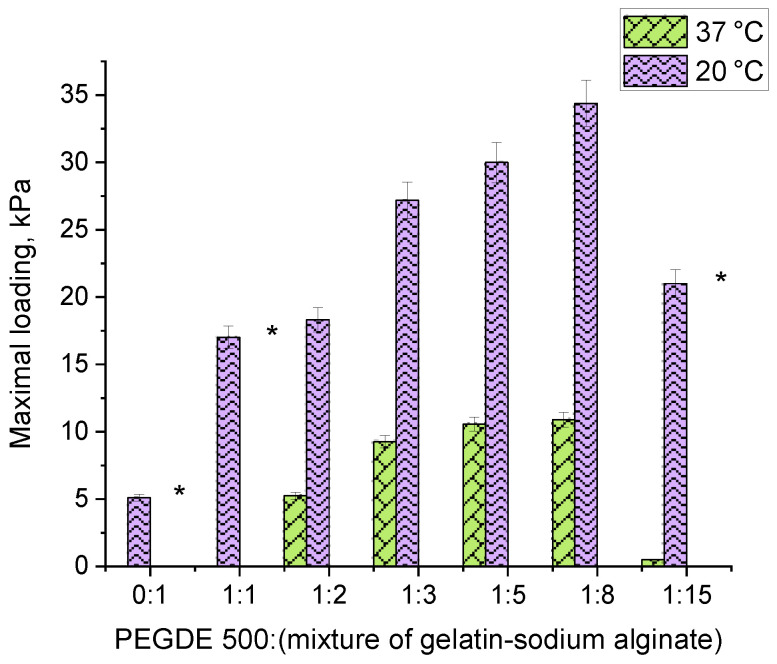
The force transmitted by the hydrogel under uniaxial compression at a strain of 4.5 mm. (For hydrogels obtained at 20 °C *—hydrogels are destroyed under compression, and the rest of the samples remained intact).

The analysis of the force developed under the uniaxial compression of hydrogel samples (Figure 5) revealed that, as follows: (1) replacing 1/10 of gelatin with sodium alginate significantly enhances the strength of the hydrogel, increasing it from 5.1 kPa to 17 kPa, and (2) the force depends on the amount of the PEGDE 500 structuring agent in the hydrogel. A maximum force of 34 kPa is achieved at a ratio of 1:8. At this ratio, hydrogel contains a total of 12% of the gelling agent polymer with 16.66% being the structuring agent. This composition results in the maximum number of effective cross-linking nodes within the polymer network, forming a robust hydrogel framework.

These findings correlate well with the gel-fraction data, which also have a maximum value with the composition (Figure 3, ratio 1:8). It is worth noting that the elasticity of the samples varies with the PEGDE 500 content. At ratios ranging from 1:2 to 1:8, the hydrogels withstand a maximum deformation without destruction at both 20 °C and 37 °C. In contrast, at a ratio of 1:15 (corresponding to a 6.25% structuring agent content in the gelling polymer), the hydrogel exhibits a similar mechanical behavior to unstructured gelatin, becoming destructed upon reaching a specific deformation threshold (slightly higher than that for intact gelatin), even at 20 °C. Thus, the optimal concentration range for the structuring agent in the hydrogel lies between 9.11% and 25%. This range produces hydrogels with improved mechanical properties. In terms of the total concentration of components in the hydrogel, this value ranges from 1.09 to 3.0%.

### 2.4. Rheological Characteristics of Gelatin–Alginate Hydrogels

**Figure 6:**
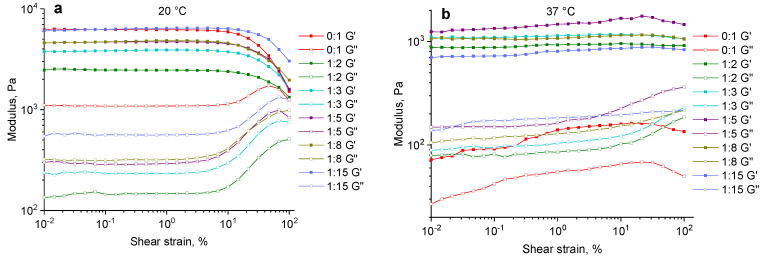
Strain (amplitude sweep) for gelatin–alginate hydrogels crosslinked with different amounts of PEGDE 500 at a temperature of 20 °C (**a**) and 37 °C (**b**).

The mechanical behavior of the hydrogels obtained from the gelatin–alginate mixture structured with PEGDE 500 was evaluated using the storage modulus (G′) and loss modulus (G″) to quantify the materials’ viscoelastic properties. Initially, an amplitude sweep was performed at a fixed frequency ω = 10 rad/s to determine the linear viscoelastic region (LVR) of each hydrogel sample. The obtained data reveal that the linear region of both the dynamic moduli (G′, G″) for all of the samples (except for the sample without PEGDE 500) at temperatures of 20 °C and 37 °C is observed in up to approximately 7% of the strain (Figure 6a,b).

**Figure 7:**
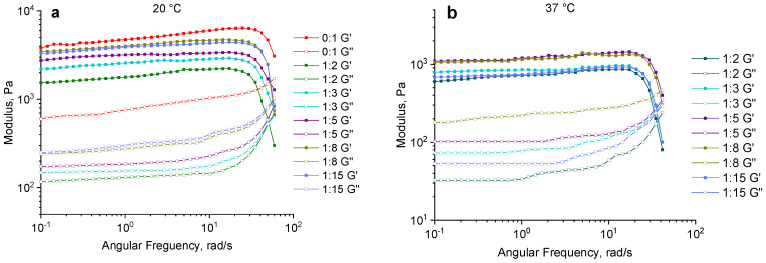
A frequency sweep for gelatin–alginate hydrogels crosslinked with different amounts of PEGDE 500 at a temperature of 20 °C (**a**) and 37 °C (**b**).

The frequency sweep was conducted by varying the angular frequency from 1 to 150 rad/s at a strain of 1%, which corresponds to the previously determined LVR for all of the samples and temperatures (Figure 6). The dependence of the dynamic moduli on the angular frequency shows a characteristic response of a well-developed elastic polymer network, with the storage modulus (G′) being significantly higher than the loss modulus (G″) (Figure 7).

The independence of G′ and G″ values from the angular frequency (ω) in the range of 0.1–10 rad/s, both at 20 °C and 37 °C, indicates the presence of a well-structured polymer network that forms a hydrogel. However, the effect of the structuring agent content on these moduli shows an unclear dependence at different temperatures. At 20 °C, the storage modulus has maximum values of up to 3000 Pa at structuring agent ratios from 1:5 to 1:15. At higher contents of the structuring agent, the value decreases to 1500–2000 Pa (Figure 7a). At 37 °C, the dependence of G′ is distinct with a maximum at the ratio of 1:5. The highest loss modulus G″ is observed at a ratio of 1:8 (Figure 7b).

**Figure 8:**
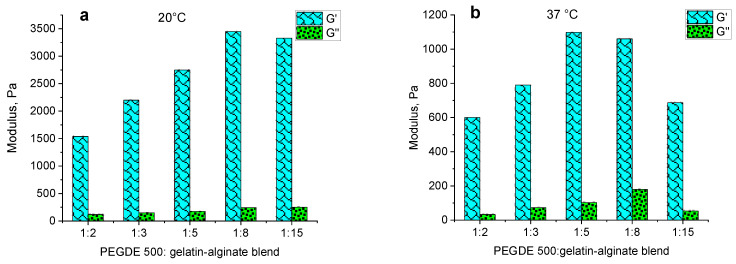
The viscoelastic characteristics of gelatin–alginate hydrogels crosslinked with different amounts of PEGDE 500 at a temperature of 20 °C (**a**) and 37 °C (**b**).

Multicomponent composites containing gelatin as the main component typically have thermoreversible properties, resulting in a significant decrease in their mechanical properties with increasing temperature. Figure 8 illustrates the dependence of the dynamic modulus of hydrogels on the content of the structuring agent. The data show that at 20 °C, the storage modulus increases as the crosslinker content decreases, demonstrating maximum values at ratios of 1:15 and 1:8. At a temperature of 37 °C, while this general trend is maintained, the optimal range is shifted towards a higher PEGDE 500 concentration, with the peak values observed at ratios of 1:5 and 1:8. Thus, the observed behavior with increasing temperatures from 20 °C to 37 °C aligns with the previously reported gelatin hydrogels, leading to a decrease in the storage modulus G′, which reflects the material’s elastic properties. This reduction in G′ ranges from a 2.6-fold decrease at a structuring agent ratio of 1:2 to a 4.8-fold decrease at a ratio of 1:15.

**Figure 9:**
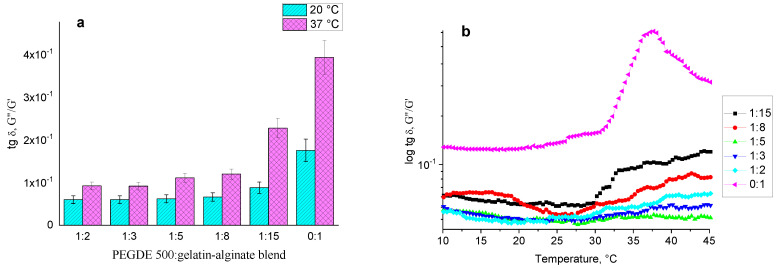
The loss tangent (mechanical loss coefficient tg δ = G″/G′) of hydrogels at different ratios of the structuring agent PEGDE 500 to the gelatin–alginate blend at 20 °C and 37 °C (**a**). The dependence of the mechanical loss coefficient on temperature for hydrogels with varying ratios of the structuring agent PEGDE 500 to the gelatin–alginate blend (**b**).

The mechanical loss coefficient calculated at a strain of 1% (Figure 9a) at 20 °C remains stable within the ratios from 1:2 to 1:8. However, at a ratio of 1:15, the coefficient starts to increase. At this ratio, corresponding to 6.25% of the PEGDE 500 in an anhydrous mixture of gelling agents, the mechanical loss coefficient is two times lower than that of an unstructured gelatin–alginate hydrogel at the same total polymer concentration (12%). At 37 °C, a slight increase in the tg δ value is observed, starting at a ratio of 1:5. At a 1:15 ratio, the coefficient increases approximately 2.5 times, which indicates the significant effect of the increased gelatin content and the decrease in elastic properties inherent for this biopolymer at higher temperatures. The temperature sweep reveals that, up to 30 °C, the mechanical loss coefficient for all hydrogel ratios is similar, but significantly lower than that of the unstructured gelatin–alginate hydrogel (Figure 9b). As was reported previously, at temperatures above 30 °C, specifically at 37.5 °C, a pronounced maximum is observed, indicating the transition of an unstructured material from a viscoelastic to a viscous flow state. This transition does not occur in the structured gelatin–alginate hydrogels, even at the lowest content of the structuring agent. Additionally, there is a decrease in the value of the mechanical loss coefficient with an increasing content of PEGDE 500 in the range of ratios from 1:15 to 1:8. At higher PEGDE 500 contents (1:2 to 1:5 ratio), the hydrogel retains its elastic properties over the entire temperature range with the mechanical loss coefficient remaining almost independent of both the ratio and the temperature.

The developed material can be promising for the creation of hydrogel dressings for wound care, so it is important to assess its performance within temperature changes from storage to use.

For the hydrogel samples, as well as for the unstructured gelatin–alginate blend (a gelatin–alginate ratio of 9:1), a decrease in both of the dynamic moduli (G′ and G″) with increasing temperature is observed, which is typical for a hydrogel of unstructured gelatin. At a higher temperature, the number of binding sites responsible for the formation of a gelatin-based gel (a gel of the 2nd kind) decreases. This reduction leads to a mechanically weaker gel, i.e., with decreased elastic moduli.

**Figure 10:**
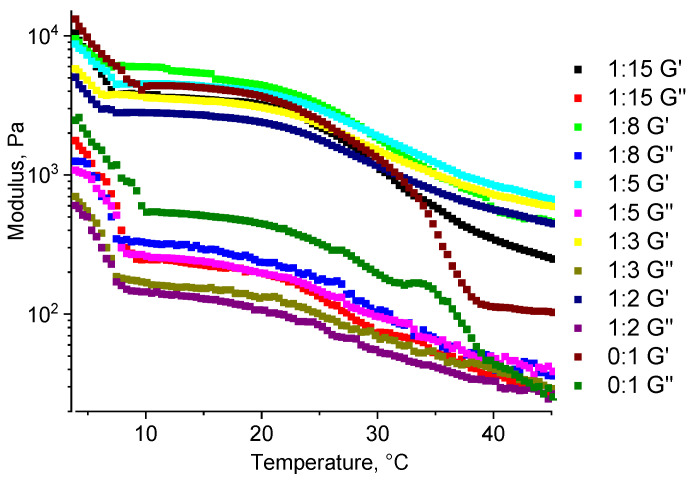
A temperature sweep for gelatin–alginate hydrogels at different ratios of the structuring agent PEGDE 500 to the gelatin–alginate blend.

The hydrogel sample without a structuring agent demonstrates the strongest temperature-dependent property change, as at 37 °C it becomes a viscous liquid (Figure 10). In contrast, for chemically structured samples, this decrease is slower, and even at 45 °C they still retain the properties of a solid (unlike liquids for which the moduli are identical). At the minimum ratio of 1:15, a strong temperature dependence is observed, but it differs significantly from the unstructured hydrogel sample. For all of the other ratios, the behavior is similar, supporting the previously obtained results of how within the studied temperature range, all of the samples of structured hydrogels retain the properties of elastic solids.

In general, the mechanical properties of the hydrogels obtained according to the developed approach are well suited for use as wound dressings [25,28,48,49].

### 2.5. The Morphological Characterization of Gelatin–Alginate Hydrogels

**Figure 11:**
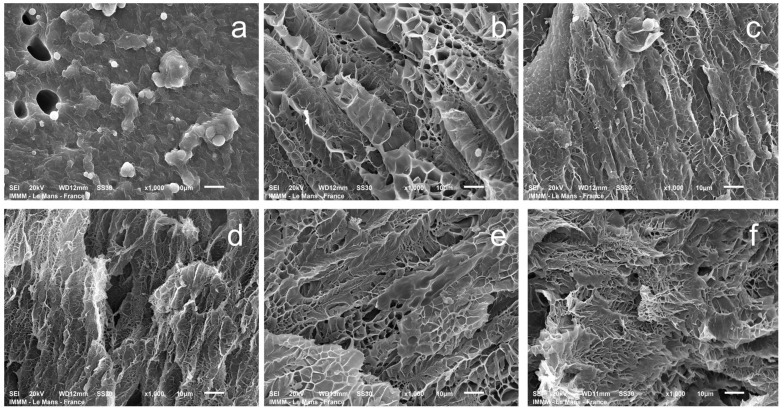
SEM images of gelatin–alginate hydrogels at different ratios of the structuring agent PEGDE 500 to the gelatin–alginate blend: 1:1 (**a**); 1:2 (**b**); 1:3 (**c**); 1:5 (**d**); 1:5 (**e**); and 1:15 (**f**), swollen twice their original size in water.

Understanding pore size is crucial for evaluating the transport properties of hydrogels and the drug release kinetics in wound healing applications [50]. Scanning electron microscopy (SEM) was used to visualize the hydrogel structure, investigating hydrogels partially swollen in water for comparison. This approach was necessary because the morphology of the hydrogel polymer network in its non-swollen state cannot be effectively evaluated by SEM. The relative differences between the samples (obtained by the same methodology at a constant magnification value of the micrograph) are quite well visualized (Figure 11). The pore size of gelatin–alginate hydrogels in the swollen state ranges from 2 to 8 μm (Figures S1 and S2) for all of the samples, except for the one obtained with a crosslinker-to-gelatin–alginate base ratio of 1:1.

The hydrogels exhibit a complex spatially oriented morphology. At a ratio of 1:1, the lamellar-layered structure is observed. At other ratios, a microcellular morphology develops, with a noticeable thinning of the structure up to a ratio of 1:5. At higher ratios, it is difficult to observe much difference. This is quite natural since, according to the chosen scheme of hydrogel syntheses, when the ratios are above 1:5, there is no significant change in the concentration of the structuring agent in the system (Table 1).

### 2.6. Swelling Capacity in Model Media

Table 2 shows the swelling degree of the obtained hydrogels as a function of the structuring agent content and temperature of the medium. These values are commonly used in the literature to characterize and predict the behavior of hydrogels as wound dressings. The general trend observed is an increase in the swelling degree with increasing temperature and with a decreasing amount of structuring agent.

For most media at 20 °C and 37 °C, hydrogel samples synthesized with the maximum amount of structuring agent, swelling similarly within the margin of error. The decreasing of the structuring agent content leads to a change in the swelling capacity but these changes vary across the different media. The most notable behavior is observed in distilled water and exudate at 20 °C and 37 °C. At 20 °C, the swelling degree in water decreases symbiotically with the decreasing content of the structuring agent, which correlates well with an increase in the gel fraction of hydrogel samples. However, at 37 °C, the swelling degree increases. This can be attributed to the increased influence of gelatin, whose content is highest in sample 5 (Table 2). As was mentioned above, gelatin forms thermally reversible physical gels which are partially destroyed with increasing temperatures. In this case, the collagen-like clusters in the gelatin partially lose their intermolecular bonds, which leads to an increased swelling degree.

In saline solution, changes in the ionic strength of the medium cause minor differences in the swelling degree of hydrogel samples. These slight differences are observed with both changes in the content of the structuring agent and temperature, while in general, the swelling degree in saline is higher than in water. A similar pattern is observed in the phosphate buffer and Ringer’s solution, where there is little dependence on the hydrogel composition. The lowest swelling degree is observed in an exudate, likely due to a significant amount of calcium ions, which interact with the functional groups of both gelatin and, more significantly, sodium alginate. These interactions lead to additional ionic structuring, and, consequently, a reduced swelling degree. This effect counters the temperature-dependent changes, so, unlike swelling in water, the swelling degree in exudate at 20 °C and 37 °C remains nearly the same.

In conclusion, despite certain features, the swelling degree of the synthesized hydrogels in all of the investigated media (60–90 g of per 1 dm^2^ of hydrogel material with a thickness of 4 mm) is sufficient to absorb wound exudates and function effectively as a dressing [51].

### 2.7. The Enzymatic Degradation of Gelatin–Alginate Hydrogels

Proteolytic enzymes (proteases) are present in various types of wounds at different stages of healing. Their primary function is to degrade necrotic tissue and cleanse the wound, which is a crucial step in the healing process [52,53]. Additionally, the presence of exogenous proteins in the wound site can promote healing and significantly affect the release of therapeutic agents from drug delivery systems.

**Figure 12:**
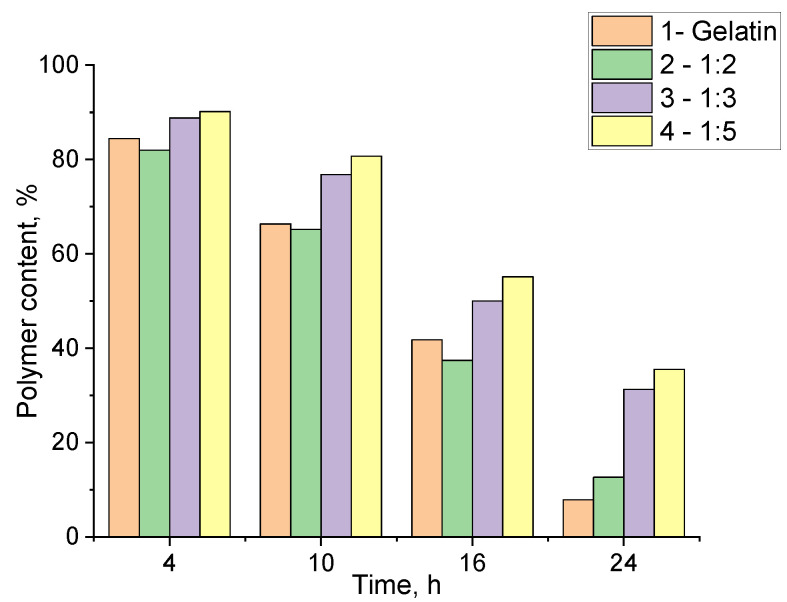
The enzymatic degradation of hydrogels at 20 °C: 1—unstructured gelatin; 2, 3, 4—gelatin–alginate hydrogels with a ratio of structuring PEGDE 500 to a gelatin–alginate base of 1:2, 1:3, and 1:5, respectively.

An investigation of the hydrogels degradation under enzymatic action is essential for assessing and predicting their behavior in vivo and in specific clinical cases [54]. The enzymatic degradation of the synthesized hydrogels was studied in vitro under model conditions using the protease enzyme (2.5 mg/mL in PBS). The percentage of residual polymers in the hydrogel was determined at specified intervals (Figure 12).

The data indicate that different structuring conditions when using PEGDE 500 have a mixed effect on biodegradation. As expected, unstructured gelatin degrades the most rapidly when exposed to proteolytic enzymes. However, the sample containing the highest amount of structuring agent also exhibited a similar degradation rate (Figure 12, samples 1,2). In contrast, hydrogels synthesized with lower ratios of PEGDE 500 demonstrated a significantly reduced degradation rate over the same time (Figure 12, samples 3,4). It can be assumed that this behavior is due to the incomplete utilization of PEGDE 500’s potential as a crosslinker in systems with its high concentrations. It appears that a significant part of PEGDE 500 interacts with the amino groups in the gelatin via only one epoxy group to form comb-shaped macromolecules with PEG chains as side substituents. This results in a reduced crosslinking degree, leading to the lower gel-fraction values and accelerated enzymatic degradation, as proteases can more effectively target the gelatin fragments, breaking down the three-dimensional network of the hydrogel.

The ability to control the degradation rate of hydrogels within specific limits could be advantageous for the development of hydrogel dressings with controlled degradation properties. Hydrogels that degrade more slowly can also be used for a prolonged, degradation-mediated release of therapeutic agents over an extended period [55]. In addition, the release of protein macromolecule fragments from gelatin into the wound can stimulate wound cleansing, thereby promoting tissue regeneration and accelerating the healing process [56,57].

### 2.8. A Study of Drug Release

**Figure 13:**
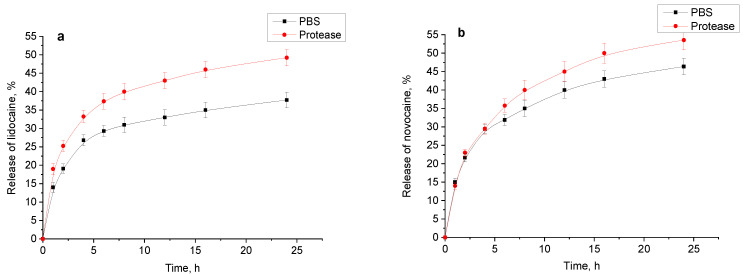

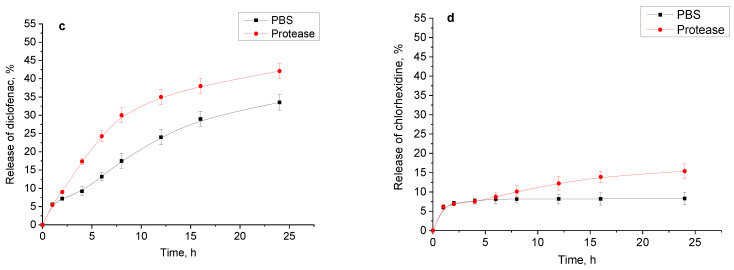
Kinetics of the drug release into phosphate-buffered saline (PBS) and into a 0.001% protease solution in PBS at 37 °C: lidocaine hydrochloride (**a**); novocaine (**b**); diclofenac sodium salt (**c**); and chlorhexidine digluconate (**d**).

The release of drugs from hydrogels was evaluated using a vertical Franz cell and hydrogel sample 4 (preparation conditions are specified in Table 1) saturated with the corresponding drug. The resulting release profiles exhibit patterns (Figure 13a,b). For lidocaine and novocaine, the typical initial rapid release for such systems was observed [25]. During this phase, 25–30% of the drugs were released, then the process slows down and 35–50% were released over the next 24 h. The presence of the proteolytic enzyme appears to accelerate the release of these drugs after the initial rapid phase, likely due to the gradual degradation of the hydrogel.

The release profile of diclofenac sodium without protease in the medium demonstrates an almost perfectly linear direct proportional dependence. However, the presence of protease accelerates the release, slightly disrupting this “ideal” release pattern. Given that the release experiments were performed using a Franz cell, the enzyme had the access to only one side of the sample. Therefore, while the enzyme’s impact was significant, it was not as pronounced as expected based on the previous section data.

For the antiseptic drug chlorhexidine, the hydrogel exhibited a limited release, achieving only 7.5% within the first 3 h, with no further increase observed within the experimental error margin. However, in the presence of the enzyme, the release continued, indicating that the release of chlorhexidine is controlled exclusively by the enzymatic degradation of the hydrogel polymer matrix (Figure 13d).

### 2.9. Antibacterial Properties

The low release capacity of the hydrogel for chlorhexidine prompted further investigation into its direct antimicrobial efficacy against pathogenic microorganisms via direct contact with the contaminated substrate. For this purpose, chlorhexidine was loaded into hydrogel disks with a diameter of 5 mm and a thickness of 2 mm. The samples were placed on the medium contaminated with *Escherichia coli* (*E. coli*), *Staphylococcus aureus* (*S. aureus*), *Pseudomonas aeruginosa* (*P. aeruginosa*), and *Aspergillus niger* (*A. niger*). The zone of microbial growth inhibition was measured to evaluate the hydrogel’s antimicrobial activity. The choice of these strains in antimicrobial studies is due to their importance as model organisms for different categories of infections: bacterial (gram-positive and gram-negative) and fungal. This allows for a more comprehensive assessment of the effectiveness of antimicrobial agents. These types of strains are very common in hospitals, in particular, *S. aureus* and *P. aeruginosa*, and cause hospital-acquired infections, which is a serious healthcare problem.

The experimental results showed that chlorhexidine loaded in the structured gelatin–alginate hydrogels can be effectively released into the contaminated substrate. This is confirmed by the significant zones of the growth inhibition of *E. coli* and *S. aureus* (Table 3). Depending on the type of bacteria, the radius of the growth inhibition varies. For *E. coli* the radius of the growth inhibition is from 12.9 to 16.5 mm, for *S. aureus* from 7.3 to 9.8 mm, for *P. aeruginosa* from 7.5 to 8.5 mm, and for *A. niger* from 1.2 to 3.6 mm.

## 3. Conclusions

The results of studies on obtaining gelatin–alginate hydrogels structured with polyethylene glycol diglycidyl ether (PEGDE 500) are presented. The conditions for synthesis were established, and the optimal ratio of the biopolymers and structuring agent in the hydrogel matrix was determined to produce materials with high strength values while maintaining the swelling capacity in water, saline, phosphate-buffered saline, Ringer’s solution, and exudate. The introduction of sodium alginate into the hydrogels, compared to the unstructured gelatin hydrogel, had a positive effect on the material properties, notably allowing for the retention of physico-mechanical properties while reducing the prepolymer content in the initial composition from 18% to 12%, and simultaneously increasing the resistance of the samples to enzymatic degradation.

The possibility of loading the samples with various medications (lidocaine, novocaine, and sodium diclofenac) was demonstrated, and the release kinetics of these drugs were studied. The results of the experiments indicate that drugs are released from the gelatin–alginate hydrogel matrix, which can provide an anesthetic effect for patients requiring pain relief.

The antibacterial properties of the hydrogels with incorporated chlorhexidine were confirmed in cultures of *E. coli*, *S. aureus*, *P. aeruginosa*, and *A. niger*, which may provide a prolonged antiseptic effect to prevent infectious complications in non-healing wounds. The obtained hydrogel material has the potential to serve as a basis for dressings in the care of chronic and/or infected wounds.

Future studies, especially in vivo experiments, are necessary to validate the findings and ensure their practical applicability.

## 4. Materials and Methods

### 4.1. Materials

Gelatin (type A), isolated from porcine skin (CAS Number: 9000-70-8, Sigma-Aldrich, St. Louis, MO, USA) was used without additional purification, with a moisture content of 9.2%, isoelectric point~8, and Bloom’s power~175. Sodium alginate (CAS Number: 9005-38-3, Sigma-Aldrich) was used without further purification. Poly(ethylene glycol) diglycidyl ether (PEGDE 500, CAS Number: 26403-72-5, molecular weight: 500, Sigma-Aldrich), was used without further purification. Lidocaine hydrochloride, purity ≥ 98% (CAS Number: 6108-05-0, molecular weight: 288.81, Sigma-Aldrich), was used without further purification. Novocaine (synonym—procaine (hydrochloride)), purity ≥ 98% (CAS Number: 51-05-8, molecular weight: 272.8, Cayman Chemical Co., Ann Arbor, MI, USA) was used without further purification. Diclofenac sodium salt (CAS Number: 15307-79-6, molecular weight: 318.13, Sigma-Aldrich) was used without further purification. Chlorhexidine digluconate solution, 20–25%, in water (CAS Number: 18472-51-0, Sigma-Aldrich) was used without additional purification, and the dry matter content was checked before use. Model exudate—a solution with pH = 7.4–7.5, was prepared according to the following procedure [58]: 0.222 g of CaCl_2_, 2.338 g of NaCl, 0.968 g of TRIS (2-Amino-2-hydroxymethyl-propane-1,3-diol), and 2 g of 5% aqueous bovine albumin solution (BSA) were added to a 100 mL volumetric flask; the salts were dissolved with stirring and made up to the mark with distilled water; and the resulting solution was stored in the refrigerator at 2–4 °C and was suitable for use within 7 days. The saline–sodium chloride solution, 0.9%, in water (CAS Number: 7647-14-5, Sigma-Aldrich) was used without additional purification. Phosphate buffered saline (PBS, Sigma-Aldrich) powder was used to prepare the aqueous solution with pH = 7.4. Ringer tablets (Sigma-Aldrich) were used for the preparation of Ringer’s solution. Distilled water, with a specific electrical conductivity of 5–7 μS/cm and pH = 6.5 ± 0.1, was used.

### 4.2. The Synthesis of Gelatin–Alginate Hydrogels

For the preparation of the hydrogel, gelatin type A, sodium alginate, the structuring agent PEGDE 500, and distilled water were used. An aqueous gelatin solution with a concentration of 20.0% and an aqueous sodium alginate solution at 4% were prepared, with each solution stirred in a water bath at 40 °C until fully dissolved. The gelatin and alginate solutions were mixed in a 10:1 ratio, respectively, based on dry matter, and the required amount of PEGDE 500 and distilled water was added. The mixture was homogenized, loaded into prepared containers, sealed, and heated at 80 °C for 4 h. This method yielded a series of hydrogel samples that varied in polymer content and component ratios.

### 4.3. Gel Fraction

The gel fraction in hydrogel samples was determined using a water extraction method. Samples weighing approximately 2.5 g were weighed to an accuracy of 0.0001 g to determine the initial mass of polymers (W_1_) in the hydrogel: W_1_ = Ws·C_p_/100, where Ws is the initial sample mass and C_p_ is the polymer content in the hydrogel (%). Extraction was conducted with a large excess (100 g of water per 1 g of hydrogel sample) of distilled water at 50 °C for 24 h, with the water changed three times. The resulting insoluble fraction was dried to a constant weight in a drying oven at 100 °C. The gel fraction (G_f_, %) was determined using the following equation: G_f_ = W_2_/W_1_·100%, where W_1_ is the mass of polymers in the hydrogel before extraction, and W_2_ is the mass of polymers remaining after extraction.

### 4.4. Fourier-Transform Infrared Spectroscopy (FTIR)

Fourier-Transform Infrared spectra were recorded using a FTIR Vertex 70V (Bruker, Billerica, MA, USA) spectrometer with the use of the Attenuated Total Reflectance (ATR) adapter (diamond crystal). The absorption bands were recorded in the range of 400–4000 cm^−1^ over 256 scans and a resolution of 2 cm^−1^.

### 4.5. Mechanical Properties

Mechanical properties of the hydrogels were assessed by applying a stepwise uniaxial compressive load in 500 μm increments over a contact area of 0.95 cm^2^, with continuous force measurement. The tests were conducted using standardized hydrogel samples with a diameter of 11 mm and a height of approximately 5.5 mm [28,59].

### 4.6. Rheological Characteristics of Polymer Solutions

The viscosity parameters of biopolymer solutions and their mixtures were studied using a Rheotest 2 rotational viscometer, which can measure viscosity in the range of 10^−2^ to 10^−4^ Pa·s over a shear rate range from 0.2 to 1.3 × 10^3^ s^−1^. A series of solutions was prepared at various gelatin-to-sodium alginate ratios (from 1:1 to 15:1, respectively) with a total biopolymer concentration of 1% in distilled water. The dynamic viscosity was calculated using the Equation (1):

η = (τ)/(γ˙)(1)

where η—dynamic viscosity, [cP]; τ—shear stress, [Pa]; and γ˙—shear rate, [s^−1^].

### 4.7. Rheological Characteristics of Hydrogels

#### 4.7.1. Viscoelastic Properties of Hydrogels

The testing of viscoelastic properties was performed using a Discovery HR-3 Hybrid rheometer (TA Instruments, New Castle, DE, USA) with a 20 mm parallel plate geometry. Hydrogels, prepared with a height of 2000 μm, were placed onto the Peltier plate, and the probe was lowered to create a controlled gap by applying a constant axial force of 0.2 ± 0.1 N, ensuring a consistent contact between the probe and the hydrogel. Samples were allowed to equilibrate for 120 s before testing, and all experiments were conducted at 20 °C in oscillatory mode. An amplitude sweep was first conducted from a 0.01% to 100% strain at an oscillation frequency of 1 Hz to identify the linear viscoelastic region (LVR), where stress and strain remain proportional. A strain of 0.25% was selected for evaluating the frequency dependence of the dynamic moduli, G′ (storage modulus) and G″ (loss modulus). Subsequently, a frequency sweep was performed from 0.01 to 10 Hz at a 0.25% strain, assessing the frequency dependence of G′ and G″ to characterize the viscoelastic behavior of the gelatin-derived hydrogels.

#### 4.7.2. Strain (Amplitude) Sweep

Hydrogel samples were incubated for 2 h at 20 °C and 37 °C before the measurement. The rheological characterization of the hydrogels samples was performed using an Anton Paar modular compact rotational rheometer MCR302 using a parallel plate probe (plate diameter 9.975 mm) at the corresponding temperatures (20 °C and 37 °C). A strain sweep was performed from 0.1 to 100%, and a shear strain (ramped logarithmic) was then conducted at a constant angular frequency of ω = 10 rad/s. Each sample was measured in triplicate and the results were averaged for each measured point. Th resulting curves were used to determine the linear viscoelastic region (LVR) for a further frequency sweep.

#### 4.7.3. Frequency Sweep

The hydrogel samples were incubated for 2 h at 20 °C and 37 °C before the measurement. A frequency sweep was performed using Anton Paar modular compact rotational rheometer MCR302 (Anton Paar, Graz, Austria) using a parallel plate probe (plate diameter 9.975 mm) at the corresponding temperatures (20 °C and 37 °C). The frequency sweep was done by varying the angular frequency from a 1 to 150 rad/s at 1% strain, which fits to the previously determined LVR. The frequency-dependence of the storage (G′) and loss (G″) modulus was evaluated as the indicator of the viscoelastic response of the hydrogels.

#### 4.7.4. Temperature Sweep

The hydrogel samples were incubated for 2 h at 6 °C before the measurement. The temperature sweep was performed using Anton Paar modular compact rotational rheometer MCR302 using a parallel plate probe (plate diameter 9975 mm). The temperature was ramped from 6 °C to 50 °C.

### 4.8. Scanning Electron Microscopy (SEM)

The Scanning Electron Microscope used is a JEOL, JSM 6510 LV instrument. The samples were swelling twice and frozen with liquid nitrogen, lyophilized (the materials were lyophilized in a Christ freeze-dryer alpha 2–4 LSC at −85 °C and 0.37 mbar), sectioned to visualize the hydrogel morphology, and were covered by a golden film before examination. Images of the hydrogels were analyzed using ImageJ Software Version 1.54 (National Institutes of Health (NIH) and the Laboratory for Optical and Computational Instrumentation (LOCI), University of Wisconsin, Madison, WI, USA).

### 4.9. Swelling Ability

The swelling capacity of hydrogels (SA) was measured by the conventional gravimetric method in distilled water (pH 6.5), saline (pH 7.0), and model exudate (pH 7.5). A total of 1 × 0.5 cm hydrogel samples were weighed and incubated at 20 °C and 37 °C in 50 mL of distilled water/saline/exudate/Ringer solution/PBS. After specified incubation times (1, 2, 4, 8, 12, and 24 h), excess liquid was removed, the sample was weighed, and then placed back in 50 mL to continue the incubation at the respective temperatures. The swelling of the scaffolds was calculated as the ratio of the swollen sample’s weight (W_W_ − W_P_) to the dry polymer weight (W_P_). Each measurement was performed in triplicate, and the results were averaged. The swelling ability (S_A_) was calculated using the following Equation (2):

S_A_ = (W_W_ − W_P_)/W_P_, (2)

where W_W_ and W_P_—the weight of the swollen sample and dry polymers, respectively.

### 4.10. The Enzymatic Degradation of Hydrogel Samples

The enzymatic degradation of hydrogel samples was conducted in a PBS solution (pH = 7.4) with the addition of protease (ORBAproteo P1200, ORBA Biokimya, İstanbul, Turkey). Hydrogels weighing 1–1.2 g were weighed with an accuracy of 0.0001 g and transferred into 10 mL of the protease solution at a substrate concentration of 2.5 mg/mL in PBS. They were then incubated at 20 °C in an air thermostat chamber for predetermined time intervals (3, 6, 16, and 24 h). After incubation, the samples were collected, dried, and weighed to determine the residual polymer content. The percentage of degradation was calculated using the following Equation (3):

Degradation (%) = (W_d_
*−* W_t_)/W_d_·100% (3)

where W_d_—polymer weight, [g] and W_t_—polymer weight that remains after incubation at time t, [g].

### 4.11. Drug Release Studies

Gelatin–alginate hydrogel samples were initially saturated with medication solutions as follows: lidocaine hydrochloride—55–60 mg/g; novocaine—4–5 mg/g; sodium diclofenac—20–25 mg/g; and chlorhexidine bigluconate—15–20 mg/g. The release of lidocaine, novocaine, diclofenac, and chlorhexidine from hydrogel samples (diameter ~15 mm, height ~4.0 mm, and weight ~1 g) was carried out in model environments: phosphate-buffered saline (PBS) and a 0.001% protease solution in PBS. A vertical Franz diffusion cell with a diffusion area of 3.53 cm^2^ and an acceptor compartment volume of 5 mL (PermeGear, Bechenheim, Germany) was used for the study. The hydrogel sample and release medium were separated by a highly porous regenerated cellulose membrane, 200 µm thick, with a pore size of 0.45 µm and a total porosity of 80–85% (Sartorius Stedim Biotech, Göttingen, Germany), providing unobstructed contact with the receptor medium. The experiments were conducted in triplicate at 37 °C with stirring at 480 rpm for 24 h. At specific time intervals (2, 4, 6, and 24 h), 0.2–0.4 mL samples were taken from the acceptor compartment of each sample, and an equivalent volume of aqueous medium was added to maintain absorption conditions in the system by keeping a constant initial volume. The concentration of the drugs in the collected samples was measured by UV spectrophotometry using a UV3100PC instrument; quartz cuvettes with Teflon stoppers type PCS8501, thickness 10 mm (Malvern, Malvern, UK) were used. Measurements were taken at the following wavelengths: λ = 262.8 nm for lidocaine; λ = 291 nm for novocaine; λ = 276.4 nm for diclofenac; and λ = 255.5 nm for chlorhexidine. The concentration (C, %) of the drug in the solution was determined using calibration equations: C = (Abs + 0.0063)/16.0 for lidocaine; C = (Abs + 0.0041)/653.0 for novocaine; C = (Abs + 0.001)/310.0 for diclofenac; and C = (Abs + 0.0304)/297.0 for chlorhexidine. The release degree (Rd) was determined by Equation (4) [25]:

(4) $$R_{d}=\frac{m_{dss}\times C\times m_{ss}}{m_{s}\times m_{ch}}$$

where m_dss_—the mass of the diluted sample solution, [g]; C—the concentration of the diluted sample solution [%]; m_ss_—the mass of the starting solution, [g]; m_s_—the mass of the sample, [g]; and m_ch_—the mass of the drug in the hydrogel sample, [g].

### 4.12. Antibacterial Studies

The effectiveness of the bactericidal agent chlorhexidine in the hydrogel samples was studied using the methods specified in [60]. Cultures of Staphylococcus aureus, Pseudomonas aeruginosa, Escherichia coli, and Aspergillus niger were grown on a solid medium (containing 4% glucose, 1% meat-peptone broth (MPB), and 2% agar in 100 mL H_2_O). For cultivation, 20 mL of the medium was poured into 90 mm Petri dishes, to which 2 mL of inoculum was added (ensuring that the medium temperature did not exceed 40 °C). To prepare the inoculum, microorganisms were transferred with a loop into test tubes containing 10 mL of the liquid nutrient medium and incubated for 24 h at 32 ± 2 °C. Hydrogel samples saturated with chlorhexidine were prepared with a diameter of 5.0 mm and a thickness of 2 mm, creating a chlorhexidine bigluconate concentration of 1% in the hydrogel. To study the effect of chlorhexidine on microorganism growth in the dishes, hydrogel discs containing the agent were placed on the nutrient medium with microorganisms. The inhibition zone of microorganism growth was recorded every 24 h. The area of microorganism growth inhibition (cm^2^) was determined using TotalLab TL120 Software Version 2009.

### 4.13. Statistical Analysis

All of the experiments were conducted in triplicate, and the results were reported as mean ± SD. Mean values were compared using an independent samples Student’s *t*-test, with *p*-values less than 0.05 considered statistically significant. The spectra obtained in the characterization of the hydrogels were processed using Origin 2019 Software Version 9.6 (OriginLab Co., Northampton, MA, USA).

## Figures and Tables

**Table 1:** The hydrogel composition and components ratio during PEGDE 500 crosslinking.

Sample No.	Weight Ratios		PEGDE 500 Content, %	Gelatin Content, %	Sodium Alginate Content, %	Water Content, %
	PEGDE 500	Gelatin–Sodium Alginate Blend				
1	1	1	8.571	8.571	0.857	82
2	1	2	5.625	11.250	1.125	
3	1	3	4.186	12.558	1.256	
4	1	5	2.769	13.846	1.385	
5	1	8	2.00	14.694	1.470	
6	1	10	1.500	15.000	1.500	
7	1	15	1.029	15.429	1.543	
8	1	25	0.632	15.789	1.579	
9	1	50	0.321	16.071	1.607	
10 *	0	1	0.00	16.364	1.636	
11 *	1	1	5.714	5.714	0.571	88
12	1	2	3.750	7.500	0.750	
13	1	3	2.791	8.372	0.837	
14	1	5	1.846	9.231	0.923	
15	1	8	1.224	9.796	0.980	
16	1	10	1.000	10.000	1.000	
17	1	15	0.686	10.286	1.023	
18	1	25	0.421	10.526	1.053	

* Under these conditions, the formation of a hydrogel structure was not observed at 37 °C.

**Table 2:** Swelling values after 24 h of incubation.

Sample No.	Water, g/g		Saline, g/g		PBS, g/g		Ringer Solution, g/g		Exudate, g/g	
	20 °C	37 °C	20 °C	37 °C	20 °C	37 °C	20 °C	37 °C	20 °C	37 °C *
1 (1:1)	12.8 ± 1.4	14.0 ± 1.7	13.1 ± 1.8	16.0 ± 1.6	12.2 ± 2.1	14.9 ± 0.9	12.6 ± 1.3	15.8 ± 1.5	9.8 ± 1.2	10.3 ± 1.3
2 (1:2)	12.5 ± 1.1	16.7 ± 1.5	12.6 ± 1.9	14.4 ± 1.4	11.8 ± 1.5	14.7 ± 0.8	11.4 ± 0.9	14.8 ± 1.2	9.4 ± 0.7	9.5 ± 0.9
3 (1:3)	11.2 ± 1.2	17.1 ± 1.4	11.7 ± 1.6	14.8 ± 0.9	11.5 ± 0.8	14.6 ± 1.2	11.3 ± 0.8	14.9 ± 1.3	9.3 ± 0.8	10.7 ± 1.1
4 (1:5)	9.7 ± 0.8	19.1 ± 2.2	11.2 ± 0.8	15.3 ± 1.3	11.0 ± 1.0	16.4 ± 1.4	10.5 ± 1.2	15.0 ± 1.6	8.6 ± 0.5	10.4 ± 0.7
5 (1:8)	8.7 ± 0.8	26.8 ± 2.0	10.9 ± 0.7	15.6 ± 0.8	10.1 ± 0.6	15.3 ± 1.3	10.1 ± 1.1	14.3 ± 1.2	8.4 ± 0.6	11.2 ± 0.8
0:1	13.6 ± 1.6	-	15.0 ± 2.2	-	14.4 ± 1.2	-	14.8 ± 1.5	-	10.2 ± 1.1	-

* At 37 °C, a slight dissolution of the samples in the exudate was observed after 24 h of incubation, therefore the data are given after 12 h.

**Table 3:** The types of microorganisms and growth inhibition zones due to the chlorhexidine bigluconate released from the samples * of gelatin–alginate hydrogels, cm^2^.

Microorganism Strain	Day 1	Day 2	Day 3
*Aspergillus niger*	** 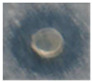 **	** 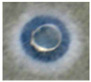 **	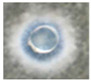
	3.45 ± 0.15	1.64 ± 0.11	1.38 ± 0.12
*Staphylococcus aureus*	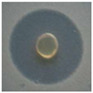	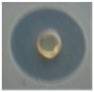	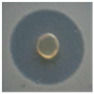
	7.65 ± 0.36	9.20 ± 0.54	9.28 ± 0.48
*Escherichia coli*	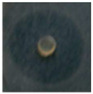	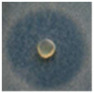	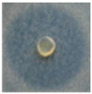
	13.60 ± 0.74	14.7 ± 0.75	15.5 ± 0.87
*Pseudomonas aeruginosa*	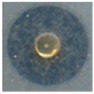	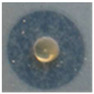	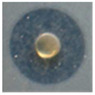
	8.29 ± 0.22	8.05 ± 0.19	7.75 ± 0.20

* hydrogel sample 3 (preparation conditions are specified in Table 1).

## Data Availability

All data and materials are available upon request from the corresponding author. The data are not publicly available due to ongoing research using a part of the data.

## Supplementary Materials

The following supporting information can be downloaded at: https://www.mdpi.com/article/10.3390/gels11030174/s1, Figures S1 and S2: Pore size of gelatin-alginate hydrogels; Figure S3: SEM image PEGDE 500-GelAl blend (1 to 1); Figure S4: SEM image PEGDE 500-GelAl blend (1 to 2); Figure S5: SEM image PEGDE 500-GelAl blend (1 to 3); Figure S6: SEM image PEGDE 500-GelAl blend (1 to 5); Figure S7: SEM image PEGDE 500-GelAl blend (1 to 8); Figure S8: SEM image PEGDE 500-GelAl blend (1 to 15).
