# Supplementary figures and images for: Functional Properties of Gelatin–Alginate Hydrogels for Use in Chronic Wound Healing Applications
Source: Gels. 2025 Feb 27;11(3):174. doi: 10.3390/gels11030174 (PMC11941921; doi:10.3390/gels11030174)

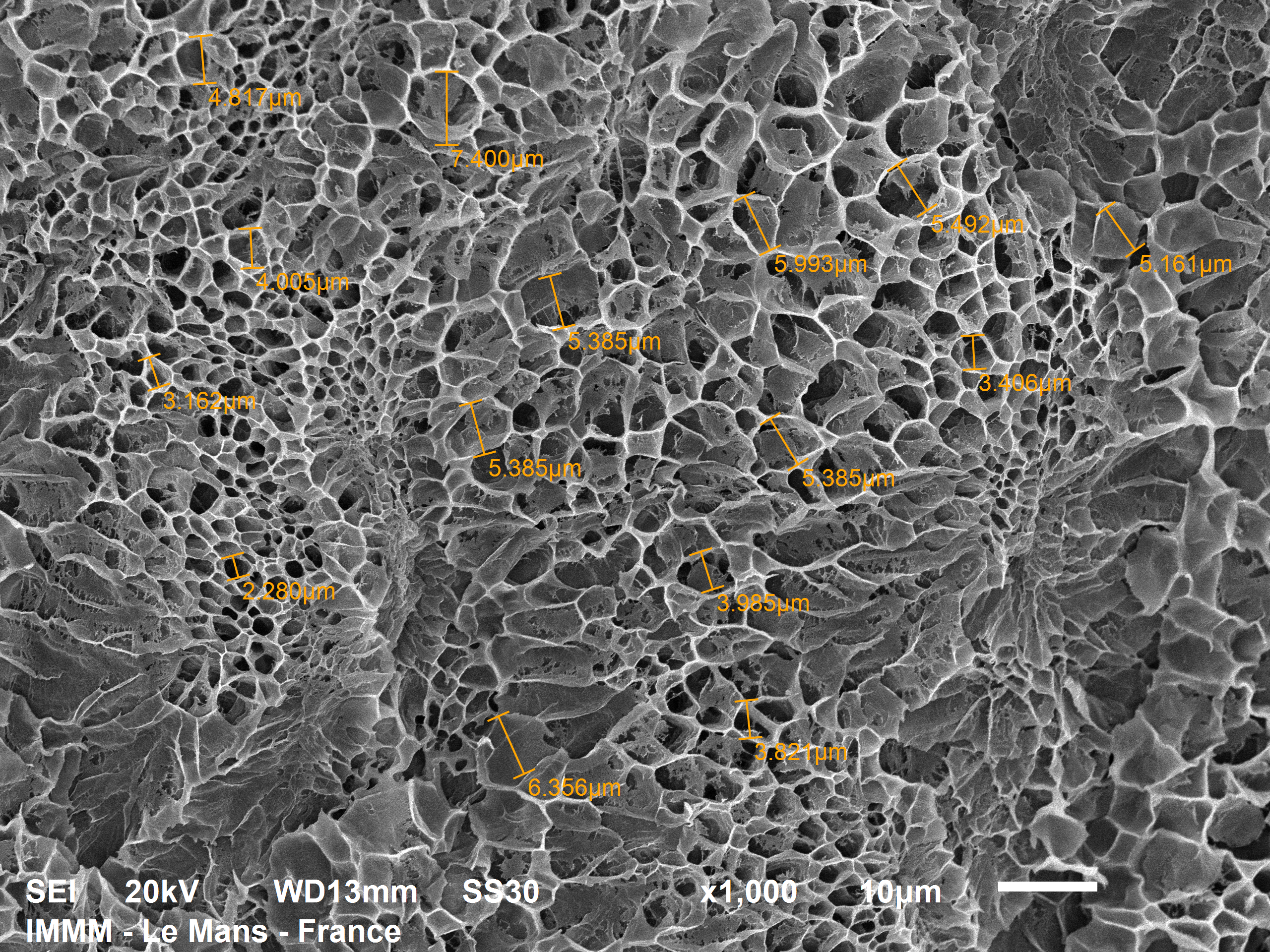

Supplement: Supplementary file 1 [file gels-11-00174-s001.zip › Figure S1. Pore size.tif]

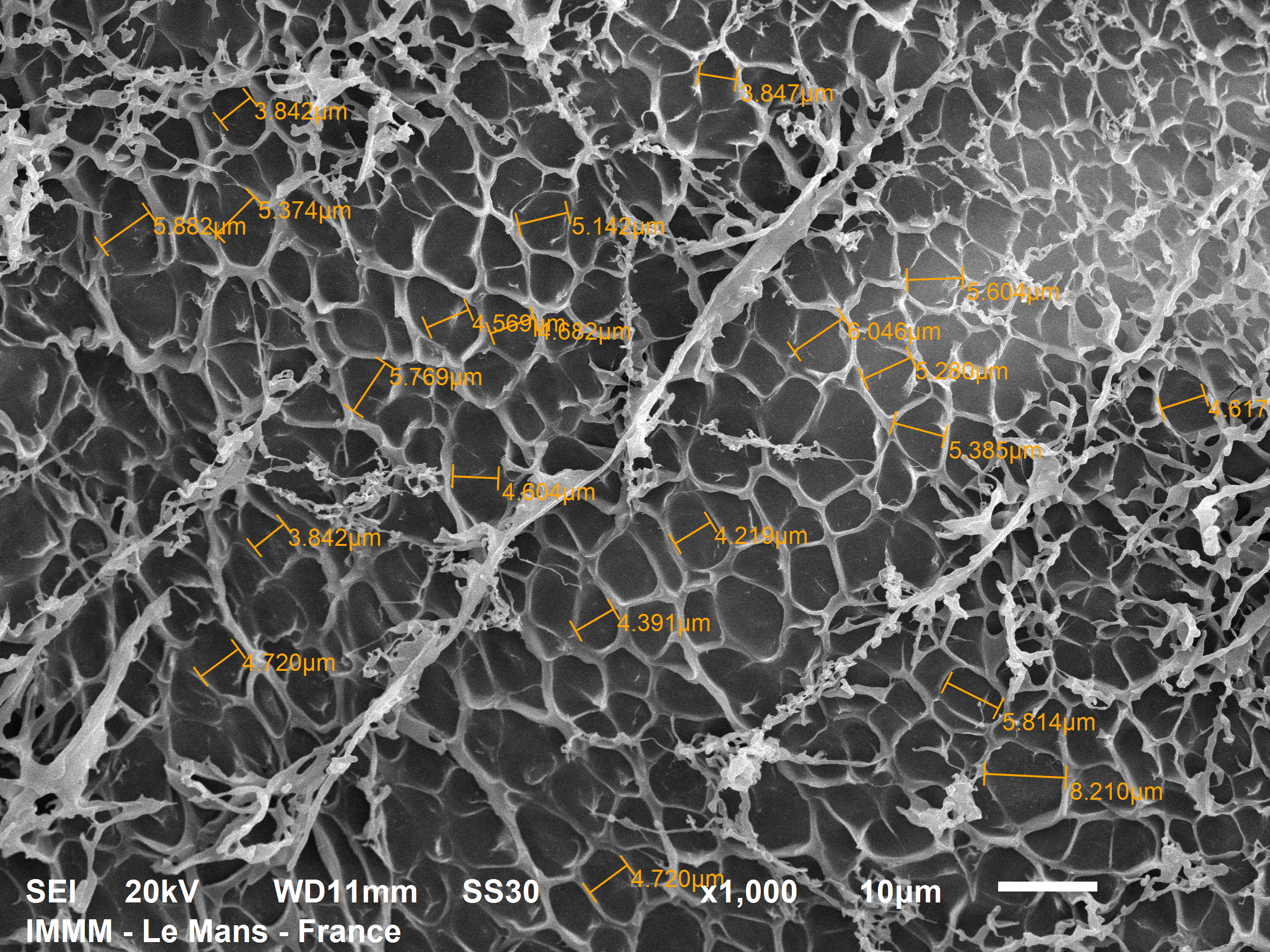

Supplement: Supplementary file 1 [file gels-11-00174-s001.zip › Figure S2. Pore size.tif]
